# The influence of the microscope lamp filament colour temperature on the process of digital images of histological slides acquisition standardization

**DOI:** 10.1186/1746-1596-9-S1-S13

**Published:** 2014-12-19

**Authors:** Anna Korzynska, Lukasz Roszkowiak, Dorota Pijanowska, Wojciech Kozlowski, Tomasz Markiewicz

**Affiliations:** 1 Nalecz Institute of Biocybernetics and Biomedical Engineering PAS, Trojdena 4 Street, 02-109 Warsaw, Poland; 2 Military Institute of Medicine, Szaserow 128 Street, 04-141 Warsaw, Poland; 3 Warsaw University of Technology, Plac Politechniki 1, 00-661 Warsaw, Poland

## Abstract

**Background:**

The aim of this study is to compare the digital images of the tissue biopsy captured with optical microscope using bright field technique under various light conditions. The range of colour's variation in immunohistochemically stained with 3,3'-Diaminobenzidine and Haematoxylin tissue samples is immense and coming from various sources. One of them is inadequate setting of camera's white balance to microscope's light colour temperature. Although this type of error can be easily handled during the stage of image acquisition, it can be eliminated with use of colour adjustment algorithms. The examination of the dependence of colour variation from microscope's light temperature and settings of the camera is done as an introductory research to the process of automatic colour standardization.

**Methods:**

Six fields of view with empty space among the tissue samples have been selected for analysis. Each field of view has been acquired 225 times with various microscope light temperature and camera white balance settings. The fourteen randomly chosen images have been corrected and compared, with the reference image, by the following methods: Mean Square Error, Structural SIMilarity and visual assessment of viewer.

**Results:**

For two types of backgrounds and two types of objects, the statistical image descriptors: range, median, mean and its standard deviation of chromaticity on a and b channels from CIELab colour space, and luminance L, and local colour variability for objects' specific area have been calculated. The results have been averaged for 6 images acquired in the same light conditions and camera settings for each sample.

**Conclusions:**

The analysis of the results leads to the following conclusions: (1) the images collected with white balance setting adjusted to light colour temperature clusters in certain area of chromatic space, (2) the process of white balance correction for images collected with white balance camera settings not matched to the light temperature moves image descriptors into proper chromatic space but simultaneously the value of luminance changes. So the process of the image unification in a sense of colour fidelity can be solved in separate introductory stage before the automatic image analysis.

## Background

This paper concerns the problem of image unification in a sense of colour and contrast as a part of projected internet platform for the automatic image analysis. This preliminary research is focused on the immunohistochemically stained with 3,3'-Diaminobenzidine and Haematoxylin (DAB&H) tissue samples. The colour's variation in samples is immense and has three origins: tissue characteristics, procedure of sample preparation and digital image acquisition stage. The diversity in tissue sample characteristics is a natural cause that cannot be eliminated. The sample preparation stage splits into the diversity of staining substances reactivity (various manufacturers), staining procedures and sample thickness (various hospital lab standards). The variability is increasing even more during the next stage, digital image acquisition, because of the multiplicity of the acquisition devices and its settings. To sum up, the images that the platform receives can be very different from each other as the digital images are coming from a variety of end-users who have various abilities and equipment to perform image capturing. The appearance variability of the digital images captured with transmitted light microscope equipped with CCD camera under various light conditions or various slides scanners makes development of effective image analysis software difficult or even impossible. Therefore, to receive reliable results, before the automatic image analysis and quantification can be done, the image unification should be performed. It is necessary to determine if the received image quality is in an acceptable range for further analysis. The images out of range should undergo the manipulation process. For example the problem of vignetting standardisation solved previously [1] can be applied if darkness of image periphery is notable in comparison to image centre. Unfortunately, any manipulation can decrease image quality in terms of colour fidelity (called also colour consistency), overall and local contrast, homogeneousness in luminance across the image, over-and underexposure, and sharpness [2]. The degrading of image is similar to effects of image compression [3]. Image features variation leads to ambiguity in contour and texture detection, thus influence reliability and effectiveness of image segmentation and image quantitative description [4]. This study concentrates on the dependence of colour variation in images captured using adequate and inadequate camera white balance (WB) setting to microscope's light colour temperature. It is important to ensure that the inadequate WB setting does not cause the loss of information responsible for unreliable results. By comparison of the images after WB correction and images which do not need this correction the range for accepted images could be developed. In this paper we also try to establish the amount of useful information wasted by inadequate WB setting and the increase done during WB manipulation.

## Methods

To find range of parameters which characterise images acceptable for automatic analysis and quantification the methodology of image comparison and image quality examination should be established. Then only images without WB settings agreement with microscopic light temperature should undergo the WB balance correction. The next subsections describe images used in this investigation, review image comparison methods and demonstrate the WB correction method.

### Captured images

**Figure 1:**
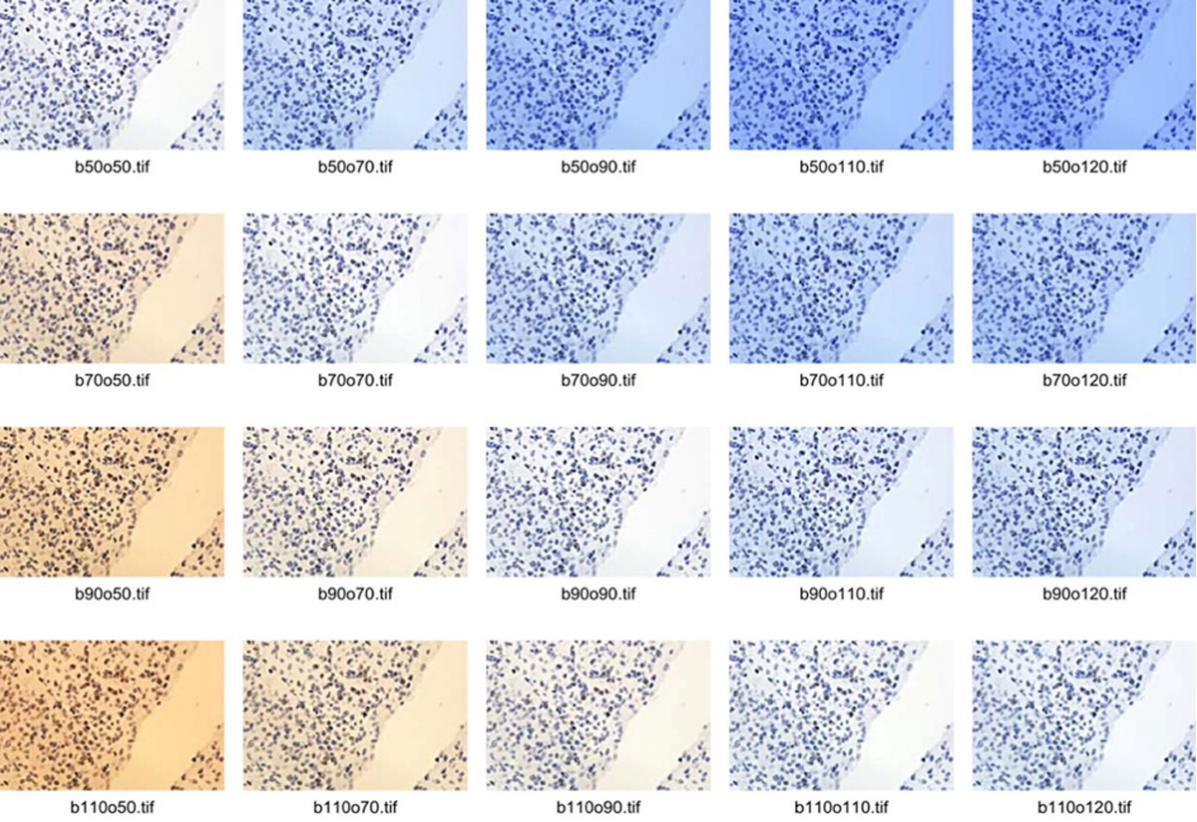
**20 images chosen from sequence of 225 images acquired with good and mismatched agreement of microscope's light colour temperature and settings of camera WB**.

The experimental material selected for image characteristics comparison has been randomly chosen from samples collected for the project of internet platform development. Six fields of view with empty space among the tissue samples have been chosen from set of tissue section slides from meningnioma patients (samples from Pathology Department of Military Institute of Medicine in Warsaw). The tissue samples have been immunohistochemically stained with DAB&H against Ki67 antigen which is nuclear protein associated with cellular proliferation and is recognised as marker to determine the growth fraction of a given cell population. Each field of view has been acquired 225 times with various microscope light temperature (15 options of lamp filament; from 0,5 up to 12 V) and camera WB settings (15 options; from 0,5 up to 12). Acquisition was done using Olympus BX61 microscope with magnification of x40 (UPlanSApo UIS2) and camera DP-72 with pixel size 0.2613 *µm *and image size 680 × 512 pixels. 20 from 225 images captured for one of 6 field of view is presented in Figure 1 to show colour diversity dependent from adequate or inadequate camera WB setting to microscope's light colour temperature. The brown objects in these images are positive nuclei during all active phases of the cell cycle (G1, S, G2, and mitosis), while blue objects are nuclei of cells resting in G0 phases.

### Methods of images descriptions and comparisons

**Figure 2:**
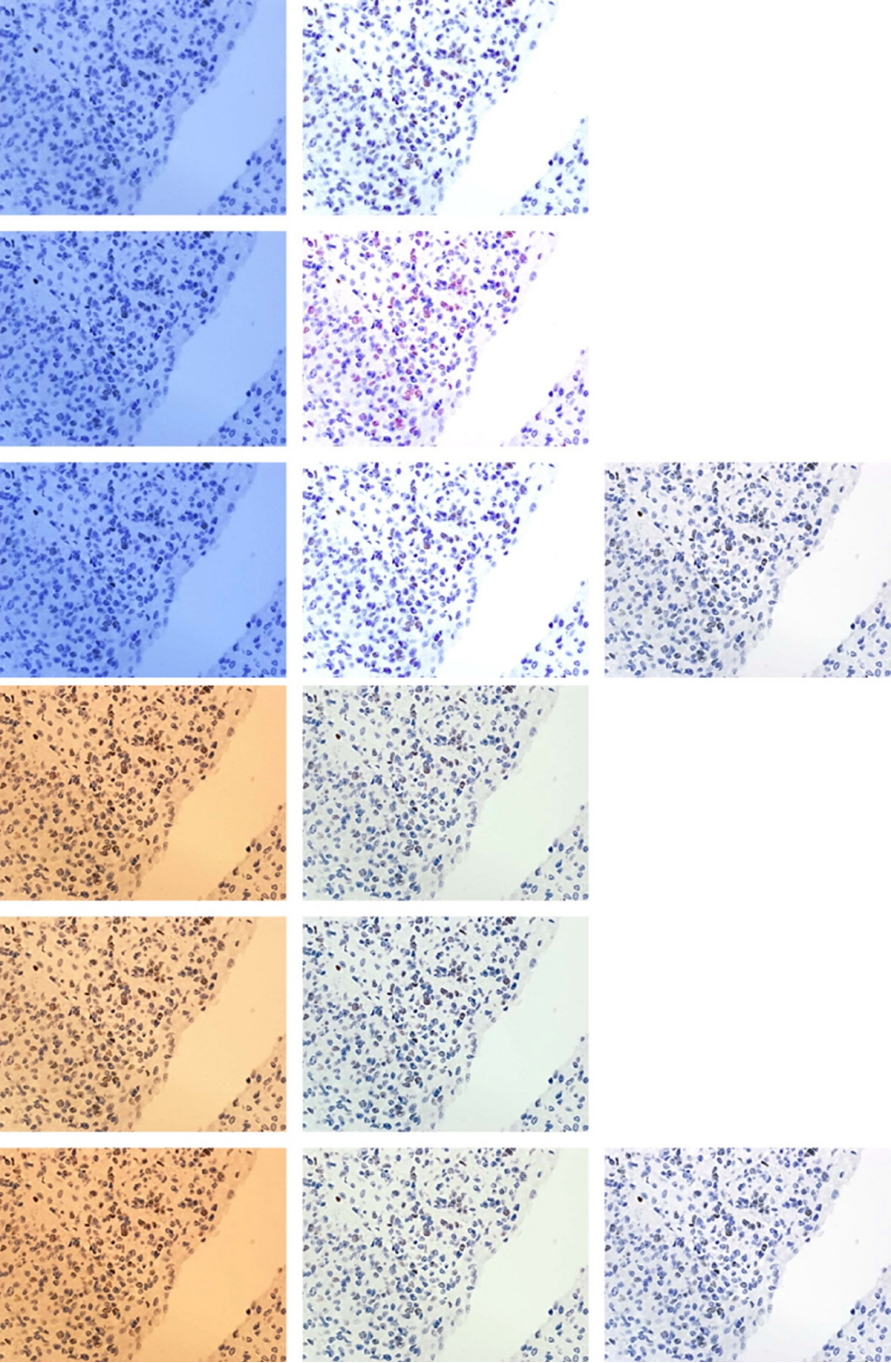
**Three most bluish and three most yellowish images from the sequence presented in Fig. 1 (left column) with results of their corrections (middle column) and reference image (right column)**.

To compare images the following methods, which measure distance between images were used; firstly Mean Square Error (MSE) [5] measures mathematical (statistical) discrepancies between two images such as sum of cumulative errors of various types and secondly Structural SIMilarity (SSIM) by Wang, Bovik [6,7] measures the similarity between two images. Both of these methods suffer from biased convergence with human judgment as they are far from human perceptual visual procedure and they require the reference image. If the reference image is not available the Commutative Probability of Blur Detection (CUBD) method proposed by the Ferzli and Karam [8] is utilizable because of its top-bottom architecture which incorporates blurring and contrast adjustment evaluation based on training database through psychophysical experiments. As any of methods is universally accepted for image quality evaluation, the comparison will be done using: MSE and SSIM methods as well as visual assessment of viewers. The statistical descriptions of images' histograms in RGB and CIELab colour models and colour variability indicated by colour range, median, mean and its standard deviation have been collected. They have been averaged in various groups (collected to resemble light and camera settings) for global spatial and local objects specific analysis. It was performed for two types of backgrounds (without tissue and with stroma) and for two types of objects - nuclei (blue-immunonegative and brown-immunopositive). In Figure 1 and 2 the clean area without tissue is shown as empty space starting from right upper corner. While, background is covered by stroma the tissue between objects is slightly tinted, almost transparent area. Both types of objects (brown and blue) are more intensively stained with inherent texture also shown in Figure 1 and 2. To use MSE, SSIM methods and visual assessment of viewers to analyse images distances the reference image for each samples was chosen. It was closest in distance to the median and mean of all images collected with adjusted colour temperature and WB settings taking in to amount chrominance and luminance variability in CIELab models. The reference image for images presented in Figure 1 and 2 is presented twice in Figure 2 in the right column.

### Method of image WB correction

**Figure 3:**
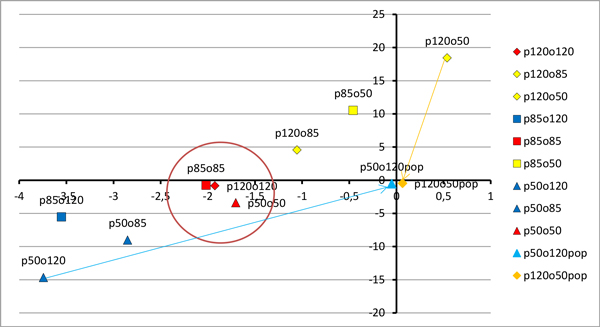
**9 images acquired with light of colour temperature 12; 8,5 and 5 V and following 12; 8,5 and 5 WB camera settings shown: in chromatic space: a - horizontal and b - vertical axis**. The 3 images acquired with lower WB setting than light temperature are marked with dark blue colour, 3 images with higher WB setting with yellow colour and 3 images with balanced WB with red colour. Images acquired with adjusted light and camera settings are located in the region marked by the red circle. The arrows connect the original and corrected image. Colour and WB manipulations causes images' colours standardization, that can be observed as orange and light blue point move closer to the (0,0) point.

**Figure 4:**
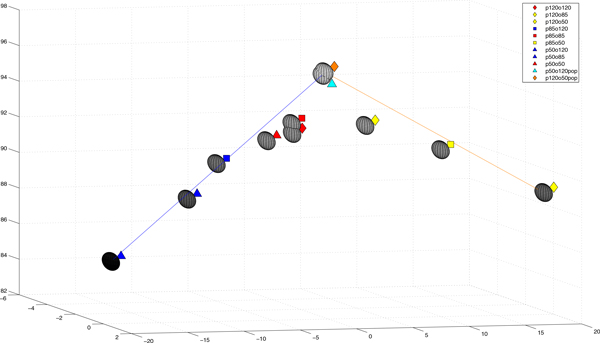
**The images from Figure 3 presented in 3D plot of Lab space: x - a, y - b, z - L**. The 3 images acquired with lower WB setting than light temperature are marked with dark blue colour, 3 images with higher WB setting with yellow colour and 3 images with balanced WB with red colour. Global lightness of image is highest for images acquired with adjusted light and camera settings while for other groups lightness is lower. The lines connect the original and corrected image. Corrected images have their lightness increased even over the level of reference image, as orange and light blue point are higher than their yellow and blue uncorrected counterpart.

Fourteen images without adjusted colour temperature and WB settings have been randomly chosen. These images have been corrected manually three times by one operator using CameraRaw 4.1 (Adobe corp.) using colour calibrated monitor under various types of light condition (sunlight, cloudy day light and bulb light). The manual WB correction, exposure correction, tonal curve manipulation has been done regarding the reference image. The WB correction was done by pointing bright pixels area in areas without tissue and gray areas with stroma. Exposure was corrected by distribution of extra light simulation according to algorithms proposed in CameraRaw 4.1. The tonal curvature manipulation has been done arbitrary to obtain range of blue colour object similar to chosen reference image. The results of colour adjustment for three most bluish and three most yellowish images in sequence presented in Figure 1 are presented in Figure 2 in the context of the reference image, while changes in Lab space are shown in Figure 3 and 4. The mathematical distances (MSE, SSIM) among corrected and uncorrected images separately are calculated to show correction efficiency.

## Results and discussion

For two types of backgrounds (clean - shown as empty space and covered by stroma - shown as grey area) and two types of objects - nuclei (immunonegative shown as blue and immunopositive shown as brown), the statistical image descriptors: range, median, mean and its standard deviation of chromaticity on 'a' and 'b' channels from CIELab colour space, as well as luminance 'L', and local colour variability have been calculated. The results have been averaged for 6 images acquired in the same light conditions and camera settings for each sample. The results for images presented in Figure 2 and 3 are presented in Table 1. It is observable that global lightness and local contrast between objects and background (both clean and covered by stroma) is higher for WB balanced acquirement, than for the others. The image with statistical descriptors closest to the averaged for all images acquired with adjustment of camera settings to the light conditions was chosen as the reference image for each sample. The reference image is used as 'gold truth' both for manual correction and for mathematical comparison. For the images presented in Figure 1 and 2 chosen reference image is shown twice in the right column of the Figure 2. To analyse loss of information on the level of acquisition with maladjustment of the camera settings to light conditions, the statistical parameters described previously have been compared. To show dependence of global image lightness on WB acquisition conditions 9 images (from the sample shown in Figure 1 and 2) and 2 corrected images are presented in 2D space of chromaticity (plane where 'a' from Lab space is on horizontal axis and 'b' on vertical axis) in Figure 3. The same images are presented also in 3D plot of Lab space in Figure 4 with vertically located L axis. In both figures there are 3 images acquired with lower WB setting than light temperature (dark blue coloured), 3 images with higher WB setting (yellow coloured) and 3 images with balanced WB (red coloured). It can be easily observed in both Figures 3 and 4 that all images acquired with adjusted light and camera settings are located in the region marked by the red circle while bluish and yellowish images are located in corners of the chromaticity space. In addition, in Figure 4, it can be observed that global lightness of image is highest for images acquired with adjusted light and camera settings while for other groups lightness is lower; lower for bluish (left part of Figure 4) than for yellowish (right part of Figure 4). When lightness is decreased the contrast between objects and background is decreasing in similar way so it was found that the global lightness and local contrast information is lower in images with maladjustment of the camera settings to light conditions than in images acquired with adjustment of WB camera setting to the light temperature. Moreover, in Figure 3 and 4 there are two extra images (light blue and orange coloured), which show results of manual correction with regard to reference image. The arrows and lines in both Figures connect the original and corrected image. It clearly shows the shift in location in the Lab space the correction provides. Both corrected images' characteristics are changed by following operations: algorithmic changes in WB on the level of colour temperature (for bluish +100; for yellowish -70 to -55), tinta (for bluish +0 to 10; for yellowish -2 to 25), selective changes in colour (for bluish in violet axis -61 to -82; for yellowish in blue 10 to 25 and violet -43 to -55), and chroma (for bluish in orange axis -11 to -29 and in blue axis 30 to 45; for yellowish in orange -33 to -45 and red 10 to 35). All these colour and WB manipulations causes that images' colours are standardized, what can be observed as both points (representing the images in chromaticity space) move closer to the (0,0) point in the chromatic space (see arrows in Figure 3). However, these manipulations do not change lightness of the image. To achieve good global and local contrast the manipulation of image contrast supported by simulation of higher expositions appears to be needed: for yellowish images additional simulated extra light should be lower than for bluish. Lastly the histogram manipulations has been made together with cutting free part of bright and dark part of histogram and increasing contrast by S-transformation. It caused the overall lightness and contrast increase. The final results have their lightness increased even over the level of reference image, as in Figure 4 where orange and light blue point are higher than their yellow and blue uncorrected counterpart. After all of the above changes the position of images characteristics moves according to the shown in Figure 3 and 4 arrows and lines. The similarity and distance between corrected and reference image in comparison to the distance and similarity between original acquired and reference image in Lab and/or RGB space agrees with results shown in Figure 3 and 4.

**Table 1:** The statistical image descriptors for images acquired in various adjustment of WB settings with light temperature.

	lower WB				adjusted WB				higher WB			
**local characteristics**	**A**	**B**	**C**	**D**	**A**	**B**	**C**	**D**	**A**	**B**	**C**	**D**

L - blue	50.20	1.71	50.54	1.63	66.69	1.25	66.96	1.05	56.60	1.93	57.14	1.84

a	14.00	1.73	14.61	1.45	3.20	1.15	4.46	2.19	13.67	1.53	13.21	1.53

b	-56.33	2.08	-56.17	2.11	-22.40	1.12	-22.29	1.10	20.67	2.08	19.78	2.25

L - brown	40.65	2.16	42.18	1.87	55.19	1.63	57.47	1.92	46.67	1.71	49.31	1.64

a	12.00	0.00	11.87	0.67	3.87	1.19	3.86	1.14	17.67	1.53	17.78	1.47

b	-45.33	1.53	-44.40	0.83	-11.73	1.03	-11.44	0.81	24.33	1.53	24.31	1.58

L - background with stroma	72.81	1.58	72.29	1.48	93.10	0.39	92.36	0.35	81.96	1.80	81.10	1.86

a	1.33	0.58	1.20	0.62	0.00	0.53	-0.05	0.44	12.67	2.52	12.95	2.25

b	-36.67	2.52	-36.88	2.30	-2.40	0.51	-2.44	1.06	34.00	3.00	33.88	2.62

L - empty background	78.04	1.41	77.74	1.54	97.83	0.20	97.53	0.20	86.80	1.63	86.47	1.64

a	-1.33	0.58	-1.26	0.27	-0.40	0.51	-0.41	0.25	9.67	2.52	9.75	2.18

b	-31.67	2.52	-31.73	2.29	0.00	0.38	0.14	0.63	34.67	2.52	34.47	2.76

global characteristics												

L	70.85	1.20	67.95	1.49	91.42	0.49	87.19	0.39	80.78	1.80	76.23	1.81

a	2.33	0.58	3.78	0.66	0.27	0.46	0.71	0.53	13.00	2.00	12.98	2.07

b	-38.00	2.00	-40.41	2.18	-3.13	0.52	-6.50	0.44	33.33	2.52	31.10	2.54

R	142.33	5.13	135.34	5.48					239.67	1.15	225.68	1.11

G	174.00	3.61	166.05	4.19					190.67	6.66	179.09	6.39

B	243.00	0.00	238.59	0.43					137.00	9.54	131.20	9.26

Local and global characteristics of color and lightness in Lab and RGB color model calculated for images acquired in various adjustment of WB settings with light temperature for the sample shown in Fig. 1 and 2 calculated as mean and standard deviation of 6 images captured in similar conditions.

Columns: A - mean of the median; B - standard deviation of the mean of the median

C - mean of the mean; D - standard deviation of the mean of the mean

## Conclusions

The aim of presented study is to compare the digital images of the tissue biopsy captured with transmitted light microscope equipped with CCD camera under various WB camera settings and light conditions. It is a part of investigation of the methodology of colour standardization. Because there is huge number of factors which influence the colour in digital images of the immunohistochemically stained with DAB&H tissue samples coming from various hospitals' laboratories, we propose to solve the problem of image unification in a sense of colour and contrast adjustment in separate introductory stage before the automatic image analysis. Introductory stage which unify microscopic cell images before analysis is typical technique in microscopic image processing problems [9-12] as microscopic images presents immense variability in appearance because of inherent features of such images formations. Described in literature introductory step of image processing produce version of microscopic images with assumed range of gray, with uniform light distribution, with increased global and/or local contrasts, or even with noise reduction what is typically needed for gray scale image analysis. However, in case of colour images introductory stage should standardize not only luminance but also colour information. As described in literature [2,13-15] the colour standardization is done by colour image decomposition into gray images which contending only chosen colour intensity information that is standardized. As information about colours is related to each other, the methodology should standardize images before extracting colour from them. The standardization process is done in several steps which are dedicated to various image characteristics.

The results of presented investigation leads to the following conclusions: (1) the images collected with white balance setting adjusted to the microscopic light colour temperature clusters in certain place of the chromatic space, (2) the process of white balance correction for images collected with white balance camera settings not matched to light temperature moves image descriptors into proper chromatic space but it strongly affects the luminance. Both conclusions show that global and local statistics of colours in Lab and RGB colour spaces for the stained with DAB&H tissue sample images indicates direction of image transformation to obtain images more similar to assumed reference image. It was shown that manual adjustment to the chosen reference image by manipulation of WB, exposure, tonal curve, colour and chroma results in colour unification on the level of visual expectation and mathematical similarity and distance measurements. Therefore, the automation of this procedure should be included in the introductory step before automatic image quantification using algorithms proposed in internet platform which are still under development.

So far we have proposed the method of light inequality in image plane elimination for digital images of the immunohistochemically stained with DAB&H tissue samples [1]. The solution for the problem of colour variability in the sense of maladjustment of WB camera settings to the temperature of microscopic light is proposed in this paper while the next aspect of colour standardization will be investigated further. The next step of investigation with artificial images of controlled degrees of colour quality will allow us to find resistance and tolerance of particular method of analysis for image quality.

## List of abbreviations

CCD: charge-coupled device; CIELab: Comission Internationale de l'Eclairage Lab color space (where 'L' stands for lightness and 'a' and 'b' are the colour-opponent dimensions); CUBD: Commutative Probability of Blur Detection; DAB&H: 3,3'-Diaminobenzidine and Haematoxylin; MSE: Mean Square Error; RGB: red, green, blue colour model; SSIM: Structural SIMilarity; WB: white balance

## Competing interests

The authors declare that they have no competing interests.

## Authors' contributions

AK conceived the study, designed the algorithms, and prepared manuscript. LR discussed the methods, implemented the algorithms, prepared manuscript and figures. DP discussed the methods and results. WK collected samples. TM discussed the methods and results, collected images. All authors read and approved the final manuscript.
